# Race, ethnicity and ancestry in global diabetes research: grappling with complexity to advance equity and scientific integrity – a narrative review and viewpoint

**DOI:** 10.1007/s00125-025-06516-1

**Published:** 2025-08-07

**Authors:** Nish Chaturvedi, Benjamin F. Voight, Jonathan C. Wells, Cheryl Pritlove

**Affiliations:** 1 Unit for Lifelong Health & Ageing, Research Department of Population Science & Experimental Medicine, Institute of Cardiovascular Science, University College London, London, UK; 2 Department of Systems Pharmacology and Translational Therapeutics, University of Pennsylvania – Perelman School of Medicine, Philadelphia, PA USA; 3 Department of Genetics, University of Pennsylvania – Perelman School of Medicine, Philadelphia, PA USA; 4 Institute for Translational Medicine and Therapeutics, University of Pennsylvania – Perelman School of Medicine, Philadelphia, PA USA; 5 Population Policy and Practice Department, University College London Great Ormond Street Institute of Child Health, London, UK; 6 Li Ka Shing Knowledge Institute, Unity Health Toronto, Toronto, ON Canada; 7 Dalla Lana School of Public Health, University of Toronto, Toronto, ON Canada

**Keywords:** Equity, diversity and inclusion, Genetics, Intergenerational diabetes risks, Population groups, Population stratification, Review, Structural and environmental determinants of health

## Abstract

The global burden of diabetes—across major forms such as type 2 diabetes, type 1 diabetes and gestational diabetes mellitus—disproportionately affects people of non-European ancestry, the majority of whom live in low- and middle-income countries. The heterogeneity of diabetes risks and phenotypes indicates that knowledge derived principally from European-origin populations may not be readily transferable to other groups. In this review our aim is to enhance the quality of diabetes research by championing the inclusion of diverse populations, ensuring clarity of population definition and encouraging exploration of population differences. We review the terminology used to define populations and make recommendations on the use of these terms. We argue that population membership by itself does not determine risks or response to intervention; rather, it is the confluence of genetic, environmental, sociocultural and policy factors that are causal and should be identified. We note that, while common diabetes forms are polygenic and populations are unlikely to harbour single genes that account for significant risk, environmental change that impacts lifestyle and biology demonstrably alters diabetes risk and provides opportunities for effective intervention. Similarly, while genetic variants are associated with adverse events, population group membership may sometimes not be a valid proxy for such variants, which has implications for healthcare equity. For most drugs used in diabetes there is little evidence that drug responsiveness materially differs by population grouping, although it is only recently that well-designed studies have been performed. In contrast, other population characteristics, such as sex, age and obesity, appear to alter glucose-lowering drug effectiveness and should be considered when prescribing. Inclusion of diverse populations in diabetes research, combined with a multidisciplinary approach, is essential if we are to combat the global burden of diabetes.

## Introduction

There are marked differences between populations in the risks of all major forms of diabetes (type 2 diabetes, type 1 diabetes and gestational diabetes mellitus [GDM]) [1–3]. Whereas incidences of type 2 diabetes and GDM are highest in people of Asian, African and Latin American descent, the incidence of type 1 diabetes is highest in people of European descent, living largely in Europe and North America. However, these latter regions represent only about 14% of the global population, while Asia accounts for approximately 60% and Africa around 20% of the global population [4]. Thus, the numbers of people affected by each of the major forms of diabetes are, and will increasingly be, greater in low- and middle-income countries (LMICs). At the same time there are increasing economic and geopolitical challenges, which may compromise efforts to prevent and treat disease in areas where the need will be substantial [5]. This has serious implications for global public health and healthcare systems [6].

With notable exceptions, for example the Indian Diabetes Prevention Programme [7], much of our current understanding of the causes and consequences of diabetes, and the evidence that underpins interventions to prevent and treat disease, derives from studies conducted in European-origin populations residing in high-income countries, for example the DCCT, the UK Prospective Diabetes Study and the US Diabetes Prevention Program [8–10].

But populations across the globe experience different levels and combinations of risk factors for diabetes. These in turn will result in population differences in intermediate traits, such as fat distribution and insulin resistance in the case of type 2 diabetes, thus contributing to population-level differences in diabetes incidence, disease phenotype and complication risk [11–14]. While these differences have largely been studied in the context of type 2 diabetes, similar considerations apply to understanding variations in risks and phenotypes of GDM and type 1 diabetes between populations.

The aim of this review is to illuminate the complexity underpinning the taken-for-granted term ‘population’ and encourage researchers to not only define populations with greater care, but also seek explanations for observed differences that recognise the complex interplay of biological, genetic, social, environmental and structural factors that shape population-level health. In this way we wish to catalyse and facilitate research that identifies explanations for population differences in diabetes risks and outcomes, to better enable effective interventions.

## Terms used to define populations

### Geography

Populations are often grouped geographically, by country, continent or global region, with the last favoured by international bodies such as the WHO when describing patterns of diabetes risk [1]. When investigating mechanisms and interventions, terms such as ‘race’, ‘ethnicity’ and ‘ancestry’ are employed, often interchangeably, sometimes together (e.g. race/ethnicity) and without clear definition. Rarely do researchers explicitly justify their choice of terminology or consider the implications of these classifications [15].

### Race

The term ‘race’ is a historical and sociopolitical construct, primarily based on superficial physical traits such as skin colour. A critical but erroneous assumption is that race serves as a surrogate for genetic distinctiveness, specifically that variants associated with physical characteristics that are used to define race are closely linked to those influencing disease susceptibility or drug response. This further implies that there are distinct genetic boundaries between racially defined groups. Challenges to both assumptions include the lack of supportive data, biased research studies and misleading interpretation of findings [16]. Although well recognised in the broader scientific literature [17], these insights have been given significant prominence in biomedical research by the National Academies of Sciences, Engineering, and Medicine (NASEM) consensus report on genetic and genomic research, which strongly discouraged the use of race in this context [18]. Nevertheless, papers published shortly after release of the NASEM report, including output from the US All of Us study, which used the term ‘race’ [19], illustrate the challenges faced by the research community in moving beyond this outdated and misleading framework and highlight the ongoing controversy generated by this topic [20]. Additionally, although the NASEM guidance is welcome, its narrow brief to focus on genetic research may inadvertently reinforce the primacy of genetic explanations for population differences in diabetes, overlooking the strong impact of sociostructural, environmental and policy factors in shaping diabetes risk and outcomes. In contrast, a European Union guidance note places equality as the rationale for collecting racial or ethnic data for multiple applications [21]. Before abandoning the use of the term ‘race’, we should recognise that its origins stem from the intention of categorising populations in a hierarchy of power and separation [22]. When people are categorised in terms of race, the label is a marker of exposure to that categorising process, and hence of exposure to different forms of systemic, structural and/or interpersonal discrimination, including, specifically, racism. These sociopolitical correlates of race are identified as powerful determinants of health, and key drivers of health inequalities, including in relation to diabetes [23–25]. Indeed, the NASEM report [18], although focused on genetic research, recommended retention of racial categorisation when investigating the role of racism in health.

### Ethnicity

The term ‘ethnicity’ is often preferred, and indeed is the dominant term used outside North America, although it is inconsistently defined. In contrast to race, ethnicity encompasses a broader, more fluid set of characteristics, including geography, descent, language, religion and cultural practices. It is primarily a self-ascribed identity that may change over time and according to the purpose for which such categorisation is to be used [26–28]. Crucially, ethnicity does not imply a genetic basis for individual or population differences in disease risk and incorporates the influence of environmental (including neighbourhood factors such as fast-food outlet density, green spaces and pollution) as well as sociocultural factors that determine health behaviours. Notably, ethnicity can be a basis for discrimination and often serves as a marker of differential access to critical resources such as education, healthcare, employment opportunities and social networks.

### Ancestry

The term ‘ancestry’ is most often used in genomic research, with an implicit reference to genetic ancestry, indicating that it captures relatedness. However, as highlighted in recent critiques [29, 30], genetic ancestry is frequently inferred from geography, specifically place of origin of the individual and their forbears. Place of origin merges presumed genetic homogeneity with distinct environmental and sociocultural exposures, in turn influencing health behaviours. Ancestry, then, may be more a marker of shared environmental factors than true distinct genetic lineage.

### Implications and recommendations

The use of population categories such as race, ancestry and, to a lesser extent, ethnicity in biomedical research often rests on the assumption that these terms represent discrete genetic groupings. This approach not only fails to reflect the continuous nature of human genetic variation but also obscures the significant influence of environmental, social and political factors that are patterned by geography. This misrepresentation is particularly problematic in the context of diabetes, where risk and outcomes are influenced by structural and systematic forces [25, 31, 32]. Inequities such as structural racism, colonial legacies, economic marginalisation and unequal access to healthcare and healthy food options profoundly influence who develops diabetes, how it progresses and how it is treated. Environmental exposures, such as pollution, food deserts and unsafe or poorly resourced neighbourhoods, are unequally distributed across populations. They intersect with social determinants such as education, employment, housing stability and healthcare access to determine disease risk. Political determinants of health, including health coverage eligibility and the prioritisation of certain communities in public health investments, further compound these risks. Discrimination based on physical characteristics or population membership of minority, often migrant, groups is often assumed to be solely a high-income country problem. However, discrimination, leading to inequalities, does occur within LMICs among non-migrant groups, for example the caste system in India, skin colour in Brazil and tribal groups in Africa [33–35]. By concretising categories such as race and ancestry as proxies for biology, we risk obscuring societal, political and economic causes of diabetes risk differences. And by oversimplifying the complexity of population differences and masking the structural, environmental and political determinants that drive inequities in diabetes, current practices risk entrenching rather than addressing disparities.

We recommend that population classification should be tailored to the research question [29]. As an obvious example, grouping by country or region is suited to describing the global burden of disease. We recommend, as have others [36], that the term ‘race’ be used with caution, given its sociopolitical connotations, unless, for example, the objective is to understand the impact of racism or racialised social structures [29]. Ethnicity is more informative, encompassing distinct sociocultural, lifestyle and environmental factors, and to some extent genetic factors. It should be self-assigned and, while a complex construct, the majority of people can readily align themselves to a group. In parallel, classification by ancestry should be considered. Place of origin of either self or forbears may enable more granular groupings that reflect shared environments and lifestyle. We suggest that the term ‘ancestry’ should be prefixed by ‘genetically inferred’ when referring to genetic relatedness to group populations, and ‘geographical’ when referring to place of origin.

In practice, though, regardless of the terminology used, biomedical researchers may be forced to rely on limited and imprecise data to build categories. Most studies rely on self-reported identity, drawn from predefined lists, typically adapted from national censuses. These reports are shaped by sociopolitical and historical factors and may not align with how individuals see themselves or how risk is actually distributed. Their value for historical comparisons is compromised by multiple changes in groupings used over time. These categories do not have inherent biological meaning, yet they are frequently used as surrogates for genetic difference, reinforcing misleading assumptions about population-level disease susceptibility. Additionally, an increasing number of individuals whose parents may have originated from different geographical locations and who may self-identify as belonging to multiple groups often find no suitable category within prespecified clusters by race, ethnicity or ancestry. As a result, they are frequently classified as ‘mixed’ or ‘other’ and/or excluded from analysis. This is an increasingly frequent challenge. For example, in the UK and US censuses the percentage of people classified as ‘other’ rose from ~3% and ~5%, respectively, in 2000 to ~8% and ~15%, respectively, 20 years later [37, 38]. Inclusion of mixed groups may bring unique characteristics to research, specifically a different patterning of, and correlations between, environmental, lifestyle and biological factors compared with those who do not recognise themselves as being of mixed ethnicity. As such, inclusive studies that leverage this unique diversity may help to disentangle what are otherwise highly correlated determinants in non-mixed populations, and aid detection of the key causal factors.

The following sections discuss the relative contributions of genetic and environmental factors to population differences in diabetes risk, and discuss the role of population membership in driving medication choice.

## Genes and population differences in diabetes risk: the importance of population stratification

Genetic liability can determine disease risk and is central to monogenic forms of diabetes as well as the association between specific HLA haplotypes and a substantial fraction of the increased risk of developing type 1 diabetes risk [39, 40]. In contrast to these strong risks in association with single or clustered genes, hundreds of genetic variants have been associated with type 2 diabetes and GDM, with most genomic loci conferring modest effects and collectively accounting for only a fraction of the population risk. Aggregating multiple genes to create polygenic scores for prediction or using genetic variants as instrumental variables (see Text box, Some definitions) may have some value. Moreover, recent, more inclusive genetic studies have discovered previously undetected disease-associated variants, missed because of their low frequency in European-origin populations [28]. The value therefore of studying the genetics of disease in diverse populations is that it facilitates the discovery of novel, globally applicable genetic loci that may be useful for triangulation of casual inference, identification of novel mechanistic insights and, potentially, risk prediction [28, 41].
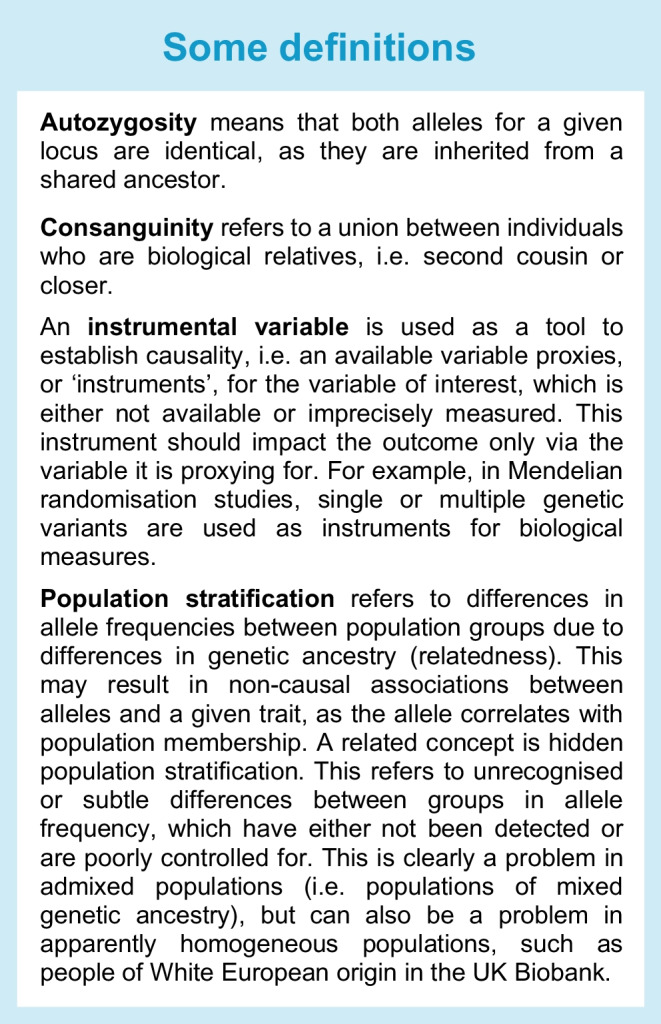


Despite success in genetic mapping, outstanding challenges remain in advancing genetic discoveries into translational outcomes that serve global health. For one, confounding due to population stratification or structure (see Text box, Some definitions) at increasingly finer resolution complicates attempts to understand the distinct risks of disease and progression to complications in personalised medicine. Systematic differences in allele frequencies across population groups can arise simply because of stochastic variation due to, for example, a combination of genetic drift and geographical isolation by distance. Associations of genetic variants with phenotype can be confounded not only when these differences in allele frequencies by population group are not taken into account, but also by health behaviours, environments and sociocultural factors that co-segregate with ancestry [42]. The association between greater levels of consanguinity (see Text box, Some definitions) and diabetes risk in some cultures is one potential example of this [43]. Inadequate sample size, adjustment only for limited socioeconomic factors (e.g. educational attainment) and imprecise measurement of, or lack of adjustment for, socioenvironmental factors challenge claims that autozygosity (see Text box, Some definitions) plays an important part in accounting for population differences in diabetes risk [44]. The extent to which hidden population stratification confounds heritability estimates or leads to incorrect attribution of genetic causality remains under-appreciated, even in apparently homogeneous populations such as the White population of UK Biobank [45].

Concerns regarding poor portability of genetic scores both between and within populations have been raised [46, 47]. This can be addressed by new statistical approaches paired with genetic risk score prediction analyses, which show that shared causal risk factors carry similar effect sizes across groups [48]. That said, the utility of these scores in clinical practice is still an open discussion [49, 50]. Further, inequalities in coverage and healthcare access will undermine the value of validated scores. Ultimately, because differences in variant frequency between populations will never account for disparities in disease risk, more attention to the complex interplay of genetic, environmental and sociocultural factors that correlate closely with population membership will be critical to uncover key mechanisms of disease.

## Environmental factors as modifiable determinants of population differences in diabetes risk: impacts of place and time

It is clear that recent secular trends in obesity map closely to those for type 2 diabetes [51] and, moreover, population differences in type 2 diabetes risk can change even within a single generation. Adult migration to settings characterised by energy-dense diets and low levels of physical activity is associated with marked and rapid increases in diabetes risk [52]. Conversely, improved environmental factors, importantly socioeconomic position, in second-generation migrants, which in turn influence diet quality and physical activity, may partially modify this increased risk [53].

While genetic factors contribute to variability in the range of phenotypic traits relevant to diabetes, these traits are also strongly influenced by environmental exposures acting through the life course and across generations, potentially with marked changes in trends over time. Global diabetes rates are closely associated with the double-burden of malnutrition; the co-occurrence of undernutrition and overweight. While these two forms of malnutrition were first observed in countries, communities or even households, they are now increasingly experienced by individuals as they pass through the life course [54, 55]. In many settings, an increasing proportion of individuals are exposed to undernutrition in early life (low birthweight, wasting and stunting) and then to overweight in later life [54]. As an example, shortness (a measure of early life stunting) coupled with adult obesity in women in India increased from 3.8% to 5.8% from 2005–2006 to 2015–2016, a relative increase of 53% [56]. Chronic exposure to undernutrition reduces body size and lean mass, while promoting adiposity to protect against food insecurity. There is little evidence that exposure to undernutrition in early life predisposes to later obesity, although it may lead to a more central distribution of body fat by constraining peripheral fat distribution. However, among those who develop overweight from childhood onwards, the adverse health effects are greater among those who also experienced poor nutrition in early life [57]. According to the ‘capacity load’ model, early undernutrition constrains the development of homeostatic metabolic capacity, a broad term referring to aspects of organ structure and function that support the maintenance of homeostasis and reduce biological ageing through the life course [58]. Through the life course, individuals are further exposed to metabolic load, another broad term referring to a range of factors that disrupt homeostasis and accelerate biological ageing [58]. Components of metabolic load relevant to diabetes include unhealthy diet, sedentary behaviour, sleep patterns, total and abdominal adiposity (overweight/obesity), smoking, inflammation and psychosocial stress. Thus, the combination of low metabolic capacity and high metabolic load is central to the emergence of diabetes across the life course. Further, this contributes to the different diabetes phenotypes observed between populations and may determine the observed intergenerational transmission of diabetes.

## Population differences in response to medication

The influence of genetic variation on drug efficacy or susceptibility to adverse reactions constitutes a specific form of gene-by-environment interaction. These interactions have been used to account for population-level differences in the response to and side effects from cardiometabolic medications, with the assumption that population group membership serves as a reliable proxy for pharmacogenetic variation [59, 60]. Examples of variation resulting in different frequencies of side effects include the threefold greater risk of angio-oedema from ACE inhibitors in people of Black African descent compared with other populations, likely in association with a greater frequency of a polymorphism in the *XPNPEP2* gene, which impacts bradykinin activity, and the similarly close to threefold excess of cough in relation to ACE inhibitors in East Asian compared with White individuals [61]. More frequently, though, population variations in side effects either are not observed or are inconsistent [62]. Variants known to influence drug response correlate both with genetically inferred ancestry and with self-identified population groupings [63]. As an example, the variant rs9923231 is associated with excess bleeding in individuals on warfarin. The T allele determining this effect is present in two-thirds of Asian participants in the All of Us study compared with one-third of White participants [63]. An important consideration raised by this study is that most pharmacogenetic research is conducted in high-income countries such as the USA and UK. Migrant populations in these settings are a highly selected subset of ancestral populations. Consequently, differences in allele frequency between migrant and host populations are likely to be exaggerated, reflecting within-migrant population relatedness, and this is one of the many ways that migrant populations living in high-income countries may differ markedly from their non-migrant counterparts. Nevertheless, even in these high-income settings, ethnic group membership does not consistently align with the distribution of pharmacogenetically relevant variants.

Associating variants with adverse events is generally more straightforward than identifying their role in drug efficacy. Characterising drug response phenotypes and separating the determinants of drug response from those influencing variability in the underlying trait remains challenging [64]. While previous pharmacogenetic studies of the effects of diabetes medication on achieving target glucose levels have been limited by suboptimal study designs [65], such as observational analyses of health records in which prescribing biases have not been accounted for, low statistical power and limited population diversity, newer studies have performed direct comparative challenges in multiple populations. These have identified variants that both associate with genetic ancestry and determine response to diabetes drugs [66]. However, currently, replication of study findings has been inconsistent, due to differences in population selection and associated population stratification and differing effects of correlated environmental factors [67, 68].

In sum, while the allele frequency of variants associated with adverse events or drug response may differ by population group, these differences are not absolute. In some cases, using population group membership as a proxy for common pharmacogenetic variants may have some utility in public health contexts, particularly when certain high-frequency alleles are strongly associated with severe adverse effects of first-line therapies [69]. Distinguishing between this ‘precision public health’ and the inappropriate use of population group membership as a proxy for pharmacogenetic alleles to drive individual precision medicine remains a challenge [70]. Clinical and demographic factors, such as age, sex and BMI, demonstrably influence drug response in diabetes in terms of achieved levels of glucose and clinical outcomes. These can be integrated into treatment decisions and provide an effective and equitable route to stratified therapy [71].

Currently, no variant so markedly influences drug response that individualised genetic testing to determine drug choice is warranted. Should this occur, it is likely that this will further drive health inequalities within and between populations.

## Conclusions and recommendations

Our recommendations are summarised in the text box. Diabetes research that overlooks population membership or focuses narrowly on individuals of European ancestry in high-income countries limits both discovery and generalisability of results. Research that transcends the traditional focus on European-origin populations is not only desirable but essential for advancing diabetes care on a global scale.
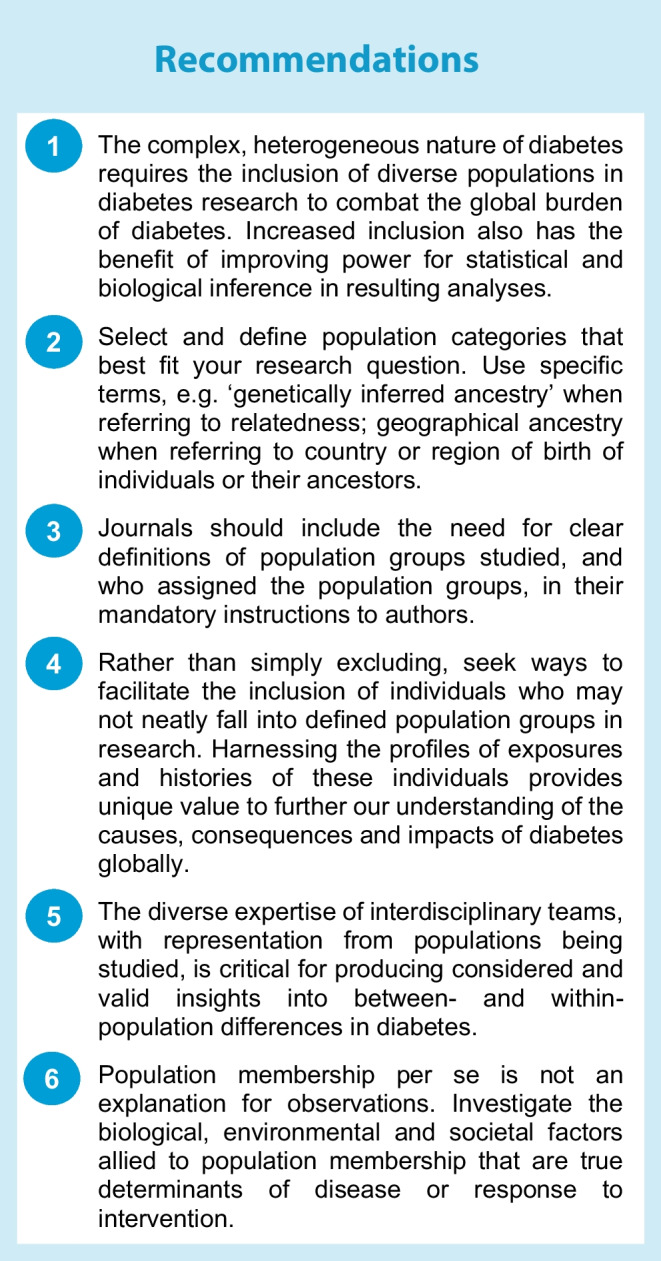


We urge researchers to consider their research question before settling on terms to categorise populations. Whatever terms are used, they must be defined clearly, including who decides on the categories assigned.

It is insufficient to simply demonstrate population differences in diabetes risks and outcomes. Population membership alone does not directly cause diabetes and its consequences. We advocate for a deeper exploration of the underlying factors contributing to these population disparities: the causes of the causes [72]. Social determinants, including poverty, food insecurity and poor access to healthcare, and the wider impacts of discrimination, acting across several generations, contribute to conditions that increase vulnerability to adverse health behaviours and outcomes, and influence receipt of optimal medication.

Additionally, most medications for diabetes and related complications work similarly across populations. Although the frequency of pharmacogenetically relevant alleles may vary by population, group membership remains an unreliable predictor of individual drug response or adverse effects.

Many factors contribute to population differences in disease risk and intervention. An interdisciplinary approach uniting biomedical and social science and including representation from the populations being studied is essential for illuminating how structural and social determinants shape health outcomes across diverse contexts. By integrating cross-disciplinary expertise, we can generate richer, more contextually informed insights that guide the development of interventions that are not only more effective, but also more equitable and responsive to the realities of those most affected by diabetes.

Finally, while much research on population differences in diabetes has focused on the overwhelming burden of type 2 diabetes, differences in type 1 diabetes and GDM should not be overlooked. Globally, the highest number of people with these conditions are not of European origin, underscoring the urgency of inclusive and global research agendas.

## Abbreviations

GDM: Gestational diabetes mellitus
LMICs: Low- and middle-income countries
NASEM: National Academies of Sciences, Engineering, and Medicine

## References

[1] GBD 2021 Diabetes Collaborators (2023) Global, regional, and national burden of diabetes from 1990 to 2021, with projections of prevalence to 2050: a systematic analysis for the Global Burden of Disease Study 2021. Lancet 402(10397):203–234. 10.1016/S0140-6736(23)01301-6 37356446 10.1016/S0140-6736(23)01301-6 PMC10364581

[2] Wang H, Li N, Chivese T et al (2022) IDF Diabetes Atlas: estimation of global and regional gestational diabetes mellitus prevalence for 2021 by International Association of Diabetes in Pregnancy Study Group’s criteria. Diabetes Res Clin Pract 183:109050. 10.1016/j.diabres.2021.109050 34883186 10.1016/j.diabres.2021.109050

[3] Green A, Hede SM, Patterson CC et al (2021) Type 1 diabetes in 2017: global estimates of incident and prevalent cases in children and adults. Diabetologia 64(12):2741–2750. 10.1007/s00125-021-05571-8 34599655 10.1007/s00125-021-05571-8 PMC8563635

[4] World Population Review (2025) Population by Continent 2025. Available from https://worldpopulationreview.com/continents. Accessed 27 Jun 2025

[5] Kehlenbrink S, Ansbro E, Besancon S, Hassan S, Roberts B, Jobanputra K (2022) Strengthening diabetes care in humanitarian crises in low- and middle-income settings. J Clin Endocrinol Metab 107(9):e3553–e3561. 10.1210/clinem/dgac331 35639997 10.1210/clinem/dgac331

[6] Non-Communicable Disease Risk Factor Collaboration (2024) Worldwide trends in diabetes prevalence and treatment from 1990 to 2022: a pooled analysis of 1108 population-representative studies with 141 million participants. Lancet 404(10467):2077–2093. 10.1016/S0140-6736(24)02317-1 39549716 10.1016/S0140-6736(24)02317-1 PMC7616842

[7] Ramachandran A, Snehalatha C, Mary S et al (2006) The Indian Diabetes Prevention Programme shows that lifestyle modification and metformin prevent type 2 diabetes in Asian Indian subjects with impaired glucose tolerance (IDPP-1). Diabetologia 49(2):289–297. 10.1007/s00125-005-0097-z 16391903 10.1007/s00125-005-0097-z

[8] Diabetes Control and Complications Trial Research Group (1993) The effect of intensive treatment of diabetes on the development and progression of long-term complications in insulin-dependent diabetes mellitus. N Engl J Med 329(14):977–986. 10.1056/NEJM199309303291401 8366922 10.1056/NEJM199309303291401

[9] Diabetes Prevention Program Research Group, Knowler WC, Fowler SE et al (2009) 10-year follow-up of diabetes incidence and weight loss in the Diabetes Prevention Program Outcomes Study. Lancet 374(9702):1677–1686. 10.1016/S0140-6736(09)61457-4 19878986 10.1016/S0140-6736(09)61457-4 PMC3135022

[10] UK Prospective Diabetes Study Group (1998) UKPDS 28: a randomized trial of efficacy of early addition of metformin in sulfonylurea-treated type 2 diabetes. Diabetes Care 21(1):87–92. 10.2337/diacare.21.1.87 9538975 10.2337/diacare.21.1.87

[11] Tillin T, Hughes AD, Godsland IF et al (2013) Insulin resistance and truncal obesity as important determinants of the greater incidence of diabetes in Indian Asians and African Caribbeans compared with Europeans: the Southall And Brent REvisited (SABRE) cohort. Diabetes Care 36(2):383–393. 10.2337/dc12-0544 22966089 10.2337/dc12-0544 PMC3554271

[12] Tillin T, Hughes AD, Mayet J et al (2013) The relationship between metabolic risk factors and incident cardiovascular disease in Europeans, South Asians, and African Caribbeans: SABRE (Southall and Brent Revisited) – a prospective population-based study. J Am Coll Cardiol 61(17):1777–1786. 10.1016/j.jacc.2012.12.046 23500273 10.1016/j.jacc.2012.12.046 PMC3677086

[13] Lear SA, Kohli S, Bondy GP, Tchernof A, Sniderman AD (2009) Ethnic variation in fat and lean body mass and the association with insulin resistance. J Clin Endocrinol Metab 94(12):4696–4702. 10.1210/jc.2009-1030 19820012 10.1210/jc.2009-1030

[14] Guerrero R, Vega GL, Grundy SM, Browning JD (2009) Ethnic differences in hepatic steatosis: an insulin resistance paradox? Hepatology 49(3):791–801. 10.1002/hep.22726 19105205 10.1002/hep.22726 PMC2675577

[15] Lu C, Ahmed R, Lamri A, Anand SS (2022) Use of race, ethnicity, and ancestry data in health research. PLOS Glob Public Health 2(9):e0001060. 10.1371/journal.pgph.0001060 36962630 10.1371/journal.pgph.0001060 PMC10022242

[16] Cerdena JP, Grubbs V, Non AL (2022) Genomic supremacy: the harm of conflating genetic ancestry and race. Hum Genomics 16(1):18. 10.1186/s40246-022-00391-2 35585650 10.1186/s40246-022-00391-2 PMC9118726

[17] Goodman AH, Moses YT, Jones JL (2019) Race: are we so different?, 2nd edn. Wiley Blackwell, Hoboken, NJ, USA

[18] National Academies of Sciences, Engineering, and Medicine (2023) Using population descriptors in genetics and genomics research: a new framework for an evolving field. The National Academies Press, Washington, DC, USA 36989389

[19] All of Us Research Program Genomics Investigators (2024) Genomic data in the All of Us Research Program. Nature 627(8003):340–346. 10.1038/s41586-023-06957-x 38374255 10.1038/s41586-023-06957-x PMC10937371

[20] Kozlov M (2024) 'All of Us' genetics chart stirs unease over controversial depiction of race. Available from: https://www.nature.com/articles/d41586-024-00568-w. Accessed 10 July 2025 10.1038/d41586-024-00568-w 38396099

[21] European Commission High Level Group on Non-discrimination, Equality and Diversity, Subgroup on Equality Data (2021) Guidance note on the collection and use of equality data based on racial or ethnic origin. European Commission, Brussels, Belgium

[22] Devakumar D, Selvarajah S, Abubakar I et al (2022) Racism, xenophobia, discrimination, and the determination of health. Lancet 400(10368):2097–2108. 10.1016/S0140-6736(22)01972-9 36502848 10.1016/S0140-6736(22)01972-9

[23] Selvarajah S, Corona Maioli S, Deivanayagam TA et al (2022) Racism, xenophobia, and discrimination: mapping pathways to health outcomes. Lancet 400(10368):2109–2124. 10.1016/S0140-6736(22)02484-9 36502849 10.1016/S0140-6736(22)02484-9

[24] Paradies Y, Ben J, Denson N et al (2015) Racism as a determinant of health: a systematic review and meta-analysis. PLoS One 10(9):e0138511. 10.1371/journal.pone.0138511 26398658 10.1371/journal.pone.0138511 PMC4580597

[25] Agarwal S, Wade AN, Mbanya JC et al (2023) The role of structural racism and geographical inequity in diabetes outcomes. Lancet 402(10397):235–249. 10.1016/S0140-6736(23)00909-1 37356447 10.1016/S0140-6736(23)00909-1 PMC11329296

[26] Phinney JS, Ong AD (2007) Conceptualization and measurement of ethnic identity: current status and future directions. J Couns Psychol 54(3):271–281. 10.1037/0022-0167.54.3.271

[27] Freeman NC, Telles EE, Goldberg RE (2025) The changing relationship between racial identity and skin color in Brazil. Proc Natl Acad Sci U S A 122(1):e2411495121. 10.1073/pnas.2411495121 39793085 10.1073/pnas.2411495121 PMC11725908

[28] Suzuki K, Hatzikotoulas K, Southam L et al (2024) Genetic drivers of heterogeneity in type 2 diabetes pathophysiology. Nature 627(8003):347–357. 10.1038/s41586-024-07019-6 38374256 10.1038/s41586-024-07019-6 PMC10937372

[29] Eastwood SV, Hemani G, Watkins SH, Scally A, Davey Smith G, Chaturvedi N (2024) Ancestry, ethnicity, and race: explaining inequalities in cardiometabolic disease. Trends Mol Med 30(6):541–551. 10.1016/j.molmed.2024.04.002 38677980 10.1016/j.molmed.2024.04.002

[30] Abdellaoui A, Dolan CV, Verweij KJH, Nivard MG (2022) Gene-environment correlations across geographic regions affect genome-wide association studies. Nat Genet 54(9):1345–1354. 10.1038/s41588-022-01158-0 35995948 10.1038/s41588-022-01158-0 PMC9470533

[31] Hill-Briggs F, Adler NE, Berkowitz SA et al (2020) Social determinants of health and diabetes: a scientific review. Diabetes Care 44(1):258–279. 10.2337/dci20-0053 33139407 10.2337/dci20-0053 PMC7783927

[32] Mujahid MS, Maddali SR, Gao X, Oo KH, Benjamin LA, Lewis TT (2023) The impact of neighborhoods on diabetes risk and outcomes: centering health equity. Diabetes Care 46(9):1609–1618. 10.2337/dci23-0003 37354326 10.2337/dci23-0003 PMC10465989

[33] Devakumar D, English S, Galappatti A et al (2025) Racisms in a global health context. Lancet Global Health. 10.1016/S2214-109X(25)00145-7 40373789 10.1016/S2214-109X(25)00145-7

[34] Reboucas P, Goes E, Pescarini J et al (2022) Ethnoracial inequalities and child mortality in Brazil: a nationwide longitudinal study of 19 million newborn babies. Lancet Glob Health 10(10):e1453–e1462. 10.1016/S2214-109X(22)00333-3 36113530 10.1016/S2214-109X(22)00333-3 PMC9638038

[35] Abdelrahim Y (2023) Tribalism among African nations: does tribalism cause oppression? Hum Nat Sci J 4:333–340. 10.53796/hnsj4521

[36] Lett E, Asabor E, Beltran S, Cannon AM, Arah OA (2022) Conceptualizing, contextualizing, and operationalizing race in quantitative health sciences research. Ann Fam Med 20(2):157–163. 10.1370/afm.2792 35045967 10.1370/afm.2792 PMC8959750

[37] Office of National Statistics (2022) Population of England and Wales. Available from: https://www.ethnicity-facts-figures.service.gov.uk/uk-population-by-ethnicity/national-and-regional-populations/population-of-england-and-wales/latest/?utm_source=chatgpt.com. Accessed 27 Jun 2025

[38] United States Census Bureau (2021) U.S. decennial census measurement of race and ethnicity across the decades: 1790-2020. Available from https://www.census.gov/library/visualizations/interactive/decennial-census-measurement-of-race-and-ethnicity-across-the-decades-1790-2020.html. Accessed 27 Ju 2025

[39] Riddle MC, Philipson LH, Rich SS et al (2020) Monogenic diabetes: from genetic insights to population-based precision in care. Reflections from a diabetes care editors’ expert forum. Diabetes Care 43(12):3117–3128. 10.2337/dci20-0065 33560999 10.2337/dci20-0065 PMC8162450

[40] Noble JA, Erlich HA (2012) Genetics of type 1 diabetes. Cold Spring Harb Perspect Med 2(1):a007732. 10.1101/cshperspect.a007732 22315720 10.1101/cshperspect.a007732 PMC3253030

[41] Lawson DJ, Davies NM, Haworth S et al (2020) Is population structure in the genetic biobank era irrelevant, a challenge or an opportunity? Human Genetics 139:23–41. 10.1007/s00439-019-02014-8 31030318 10.1007/s00439-019-02014-8 PMC6942007

[42] Tan T. Jayashankar H, Guan J et al (2024) Family-GWAS results reveals effects of environment and mating on genetic associations. MedRxiv (Preprint). 4 Oct 2024. Available from: 10.1101/2024.10.01.24314703

[43] Malawsky DS, van Walree E, Jacobs BM et al (2023) Influence of autozygosity on common disease risk across the phenotypic spectrum. Cell 186(21):4514-4527 e4514. 10.1016/j.cell.2023.08.028 37757828 10.1016/j.cell.2023.08.028 PMC10580289

[44] Johnson EC, Evans LM, Keller MC (2018) Relationships between estimated autozygosity and complex traits in the UK Biobank. PLoS Genet 14(7):e1007556. 10.1371/journal.pgen.1007556 30052639 10.1371/journal.pgen.1007556 PMC6082573

[45] Cook JP, Mahajan A, Morris AP (2020) Fine-scale population structure in the UK Biobank: implications for genome-wide association studies. Hum Mol Genet 29(16):2803–2811. 10.1093/hmg/ddaa157 32691046 10.1093/hmg/ddaa157

[46] Mostafavi H, Harpak A, Agarwal I, Conley D, Pritchard JK, Przeworski M (2020) Variable prediction accuracy of polygenic scores within an ancestry group. Elife 9. 10.7554/eLife.48376 10.7554/eLife.48376 PMC7067566 31999256

[47] Sohail M, Maier RM, Ganna A et al (2019) Polygenic adaptation on height is overestimated due to uncorrected stratification in genome-wide association studies. Elife 8. 10.7554/eLife.39702 10.7554/eLife.39702 PMC6428571 30895926

[48] Hu S, Ferreira LAF, Shi S et al (2025) Fine-scale population structure and widespread conservation of genetic effect sizes between human groups across traits. Nat Genet 57(2):379–389. 10.1038/s41588-024-02035-8 39901012 10.1038/s41588-024-02035-8 PMC11821542

[49] Abramowitz SA, Boulier K, Keat K et al (2025) Evaluating performance and agreement of coronary heart disease polygenic risk scores. JAMA 333(1):60–70. 10.1001/jama.2024.23784 39549270 10.1001/jama.2024.23784 PMC11569413

[50] Hingorani AD, Gratton J, Finan C et al (2023) Performance of polygenic risk scores in screening, prediction, and risk stratification: secondary analysis of data in the Polygenic Score Catalog. BMJ Med 2(1):e000554. 10.1136/bmjmed-2023-000554 37859783 10.1136/bmjmed-2023-000554 PMC10582890

[51] Ahmed M, Nofal A, Shafiq A et al (2025) Rising mortality rates linked to type-2 diabetes and obesity in the United States: an observational analysis from 1999 to 2022. J Diabetes Investig 16(3):492–500. 10.1111/jdi.14386 39698779 10.1111/jdi.14386 PMC11871392

[52] Kawate R, Yamakido M, Nishimoto Y, Bennett PH, Hamman RF, Knowler WC (1979) Diabetes mellitus and its vascular complications in Japanese migrants on the Island of Hawaii. Diabetes Care 2(2):161–170. 10.2337/diacare.2.2.161 520120 10.2337/diacare.2.2.161

[53] Farmaki AE, Garfield V, Eastwood SV et al (2022) Type 2 diabetes risks and determinants in second-generation migrants and mixed ethnicity people of South Asian and African Caribbean descent in the UK. Diabetologia 65(1):113–127. 10.1007/s00125-021-05580-7 34668055 10.1007/s00125-021-05580-7 PMC8660755

[54] Wells JC, Sawaya AL, Wibaek R et al (2020) The double burden of malnutrition: aetiological pathways and consequences for health. Lancet 395(10217):75–88. 10.1016/S0140-6736(19)32472-9 31852605 10.1016/S0140-6736(19)32472-9 PMC7613491

[55] Non-Communicable Disease Risk Factor Collaboration (2024) Worldwide trends in underweight and obesity from 1990 to 2022: a pooled analysis of 3663 population-representative studies with 222 million children, adolescents, and adults. Lancet 403(10431):1027–1050. 10.1016/S0140-6736(23)02750-2 38432237 10.1016/S0140-6736(23)02750-2 PMC7615769

[56] Wells JCK, Wibaek R, Poullas M (2018) The dual burden of malnutrition increases the risk of cesarean delivery: evidence from India. Front Public Health 6:292. 10.3389/fpubh.2018.00292 30386761 10.3389/fpubh.2018.00292 PMC6199394

[57] Bavdekar A, Yajnik CS, Fall CH et al (1999) Insulin resistance syndrome in 8-year-old Indian children: small at birth, big at 8 years, or both? Diabetes 48(12):2422–2429. 10.2337/diabetes.48.12.2422 10580432 10.2337/diabetes.48.12.2422

[58] Wells JCK (2018) The capacity-load model of non-communicable disease risk: understanding the effects of child malnutrition, ethnicity and the social determinants of health. Eur J Clin Nutr 72(5):688–697. 10.1038/s41430-018-0142-x 29748656 10.1038/s41430-018-0142-x

[59] Tamargo J, Kaski JC, Kimura T et al (2022) Racial and ethnic differences in pharmacotherapy to prevent coronary artery disease and thrombotic events. Eur Heart J Cardiovasc Pharmacother 8(7):738–751. 10.1093/ehjcvp/pvac040 35848895 10.1093/ehjcvp/pvac040 PMC9520447

[60] Burroughs VJ, Maxey RW, Levy RA (2002) Racial and ethnic differences in response to medicines: towards individualized pharmaceutical treatment. J Natl Med Assoc 94(10 Suppl):1–26 12401060 PMC2594139

[61] McDowell SE, Coleman JJ, Ferner RE (2006) Systematic review and meta-analysis of ethnic differences in risks of adverse reactions to drugs used in cardiovascular medicine. BMJ 332(7551):1177–1181. 10.1136/bmj.38803.528113.55 16679330 10.1136/bmj.38803.528113.55 PMC1463974

[62] Rosoff DB, Wagner J, Jung J et al (2025) Multiomic mendelian randomization study investigating the impact of pcsk9 and HMGCR inhibition on type 2 diabetes across five populations. Diabetes 1(74):120–130. 10.2337/db24-0451 10.2337/db24-0451 PMC11664021 39418486

[63] Sharma S, Marino-Ramirez L, Jordan IK (2023) Race, ethnicity, and pharmacogenomic variation in the United States and the United Kingdom. Pharmaceutics 15(7):1923. 10.3390/pharmaceutics15071923 37514109 10.3390/pharmaceutics15071923 PMC10383154

[64] Pearson ER (2024) New insights into the genetics of glycemic response to metformin. Diabetes Care 47(2):193–195. 10.2337/dci23-0060 38241501 10.2337/dci23-0060

[65] Bjornsson TD, Wagner JA, Donahue SR et al (2003) A review and assessment of potential sources of ethnic differences in drug responsiveness. J Clin Pharmacol 43(9):943–967. 10.1177/0091270003256065 12971027 10.1177/0091270003256065

[66] Li JH, Brenner LN, Kaur V et al (2023) Genome-wide association analysis identifies ancestry-specific genetic variation associated with acute response to metformin and glipizide in SUGAR-MGH. Diabetologia 66(7):1260–1272. 10.1007/s00125-023-05922-7 37233759 10.1007/s00125-023-05922-7 PMC10790310

[67] Li JH, Perry JA, Jablonski KA et al (2023) Identification of genetic variation influencing metformin response in a multiancestry genome-wide association study in the diabetes prevention program (DPP). Diabetes 72(8):1161–1172. 10.2337/db22-0702 36525397 10.2337/db22-0702 PMC10382652

[68] Wu B, Yee SW, Xiao S et al (2024) Genome-wide association study identifies pharmacogenomic variants associated with metformin glycemic response in African American patients with type 2 diabetes. Diabetes Care 47(2):208–215. 10.2337/dc22-2494 37639712 10.2337/dc22-2494 PMC10834390

[69] Jordan IK, Sharma S, Marino-Ramirez L (2023) Population Pharmacogenomics for Health Equity. Genes (Basel) 14(10). 10.3390/genes14101840 10.3390/genes14101840 PMC10606908 37895188

[70] Wilson JF, Weale ME, Smith AC et al (2001) Population genetic structure of variable drug response. Nat Genet 29(3):265–269. 10.1038/ng761 11685208 10.1038/ng761

[71] Dennis JM, Young KG, Cardoso P et al (2025) A five-drug class model using routinely available clinical features to optimise prescribing in type 2 diabetes: a prediction model development and validation study. Lancet 405(10480):701–714. 10.1016/S0140-6736(24)02617-5 40020703 10.1016/S0140-6736(24)02617-5

[72] Krieger N (2012) Methods for the scientific study of discrimination and health: an ecosocial approach. Am J Public Health 102(5):936–944. 10.2105/AJPH.2011.300544 22420803 10.2105/AJPH.2011.300544 PMC3484783

